# Nanoreactors in action for a durable microactuator using spontaneous combustion of gases in nanobubbles

**DOI:** 10.1038/s41598-022-25267-2

**Published:** 2022-12-03

**Authors:** Ilia V. Uvarov, Vitaly B. Svetovoy

**Affiliations:** 1 Valiev Institute of Physics and Technology, Yaroslavl Branch, Russian Academy of Sciences, Universitetskaya 21, Yaroslavl, 150007 Russia; 2 Frumkin Institute of Physical Chemistry and Electrochemistry, Russian Academy of Sciences, Leninsky Prospect 31 bld. 4, Moscow, 119071 Russia

**Keywords:** Nanofluidics, NEMS, Nanoscale materials

## Abstract

A number of recent studies report enhancement of chemical reactions on water microdroplets or inside nanobubbles in water. This finding promises exciting applications, although the mechanism of the reaction acceleration is still not clear. Specifically, the spontaneous combustion of hydrogen and oxygen in nanobubbles opens the way to fabricate truly microscopic engines. An example is an electrochemical membrane actuator with all three dimensions in the micrometer range. The actuator is driven by short voltage pulses of alternating polarity, which generate only nanobubbles. The device operation is, however, restricted by a fast degradation of the electrodes related to a high current density. Here it is demonstrated that the actuator with ruthenium electrodes does not show signs of degradation in the long-term operation. It is the only material able to withstand the extreme conditions of the alternating polarity electrolysis. This property is due to combination of a high mechanical hardness and metallic conductivity of ruthenium oxide. The actuator combines two features considered impossible: on-water catalysis and combustion in a microscopic volume. It provides an exceptional opportunity to drive autonomous microdevices especially for medical or biological applications.

## Introduction

Today, there are a number of reports on unusual chemical activity of aqueous interfaces for objects with a high surface-to-volume ratio^1^. Acceleration of organic reactions has been revealed in microdroplets in air^2–5^. It has been reported also that hydrogen peroxide can be formed spontaneously in microdroplets with a size from 1 to 20 μm^6^. These findings open new possibilities for biological and environmental applications, although the acceleration mechanism is still unclear. Moreover, unexplained chemical processes have been observed in bulk nanobubbles (NBs) with a size smaller than 1 μm. Shrinking air microbubbles are able to produce OH radicals without any external stimuli as was observed with the electron spin resonance spectroscopy^7,8^. It has been confirmed independently with probe molecules that OH radicals are produced by NBs filled with air, O$_2$, and O$_3$ gases^9^. The formation of free radicals is a puzzling phenomenon since no high-energy sources in the system.

Spontaneous combustion of H$_2$ and O$_2$ gases has been observed in NBs produced in a so-called alternating polarity (AP) electrochemical process, when the polarity of the electrodes alternates with a frequency higher than 20 kHz^10,11^. The heat produced by the reaction has been measured using microfluidic devices^12,13^. The normal combustion reactions in such a small volume cannot be supported, because the heat escapes too fast via the bubble walls^14,15^. The smallest bubble, where it was possible to ignite the normal combustion, had a size of 2 mm^16^. Nevertheless, the combustion proceeds spontaneously in NBs without a significant increase in the local temperature (see review^17^ for details).

The spontaneous combustion has been proposed to use as a basic principle for a new actuator to drive microdevices^12^; such an actuator can be small (with all three dimensions in the range of micrometers), fast, and strong. The most advanced piezo actuators cannot be smaller than a few millimeters to produce a reasonable stroke^18–21^. They also need a high voltage to drive them. The actuators using the electrostatic forces are weak^22–24^, but those using the thermal principle are slow^25,26^. The electrochemical actuators are notoriously slow^27–32^, since the gas can be produced fast in a closed chamber, but one needs minutes to get rid of this gas even using the electrodes with catalytic properties^33,34^. We demonstrated the electrochemical actuator that uses the spontaneous combustion of gases in NBs and has the response time comparable with that for the piezo actuators^35^. However, the principal problem of such devices is a fast degradation of electrodes. On one side, the energy deposition from exploding NBs provides significant local stresses^10,36^ on chemically inert electrodes such as Pt. On the other side, harder materials are oxidised resulting in the reduction of the current^37^ as it occurs for Ti.

In this paper we present an electrochemical microactuator that produces hydrogen and oxygen NBs in a tiny closed chamber and consumes these gases during milliseconds via the spontaneous combustion process in NBs. The device uses ruthenium electrodes, which do not show signs of degradation because of high hardness and metallic conductivity of ruthenium oxide.

## Materials and methods

### Fabrication

**Figure 1:**
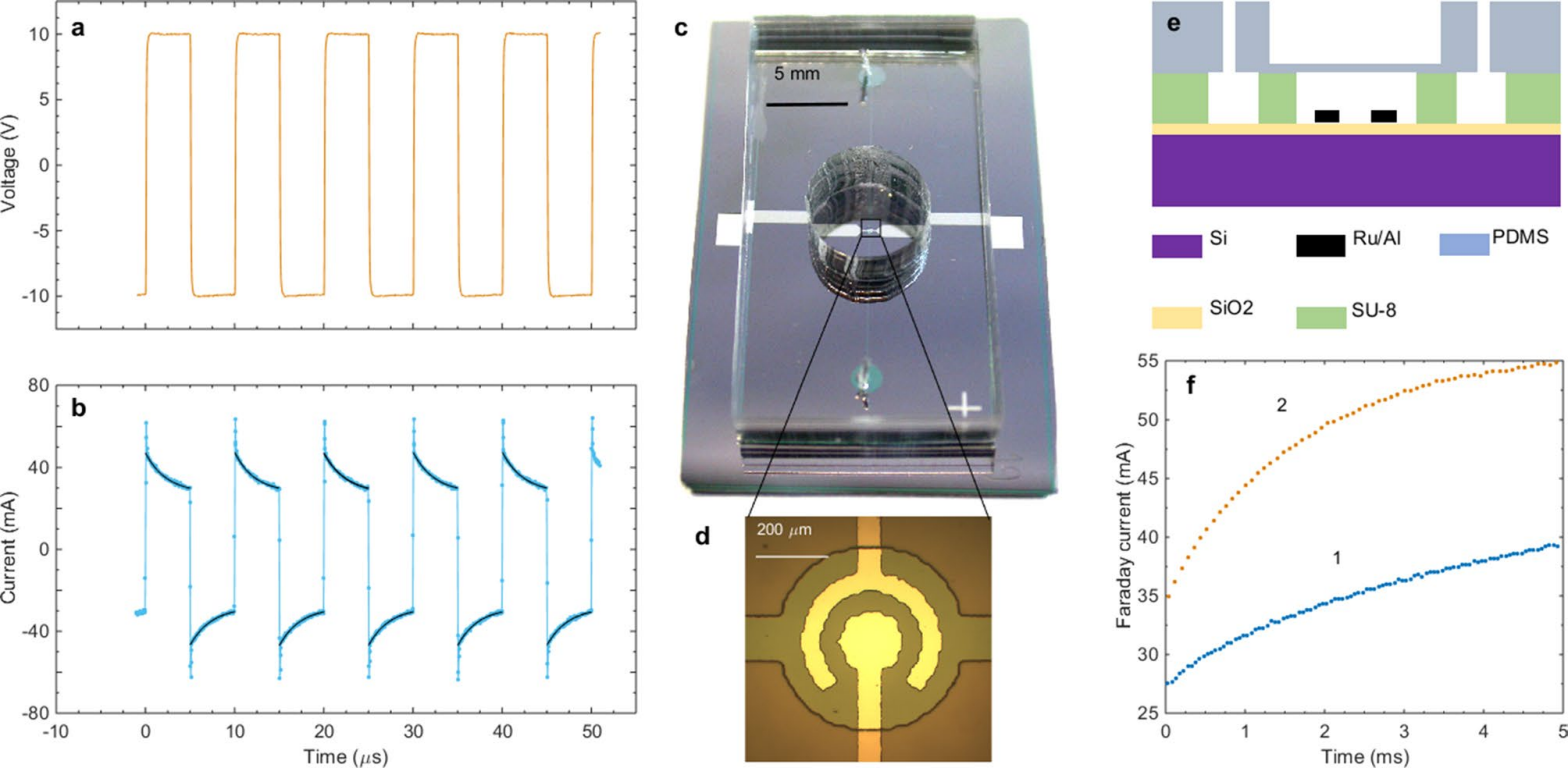
Actuator and driving pulses. (**a**) Alternating polarity voltage applied to the electrodes. (**b**) Current flowing through the electrochemical cell. For each pulse the current is described by the Faraday and charge-discharge components; the corresponding fits are shown by the black curves. (**c**) Optical image of the device; the actuator is located inside of the black rectangular. (**d**) Chamber of the actuator with the concentric electrodes. (**e**) Schematic view of the device (cross-section) showing the chamber with the electrodes covered by the PDMS membrane and openings to fill the chamber with the electrolyte. (**f**) Faraday current as a function of time for the driving frequency 100 kHz (1) and 500 kHz (2).

The actuator is fabricated on a 460 μm thick silicon wafer covered by a thermally grown SiO$_2$ layer with a thickness of 0.9 μm. For convenience of handling the chip has lateral dimensions of $20\times 35$ mm$^2$ with a working chamber in its central part as shown in Fig. 1. The diameter of the chamber is 500 μm and its height is 16 μm. The chamber includes two electrodes and is used as an electrochemical cell. The contact lines including the electrodes are made of two layers to reduce the resistance of the lines. The bottom layer is aluminum with a thickness of 500 nm. A 150 nm thick working layer of ruthenium is deposited on top of Al. The electrodes are fabricated as follows. A 10 nm thick adhesive layer of Ti is deposited first on the substrate followed by the deposition of Al (500 nm). The pattern of the electrodes is formed in the Microposit S1813 SP15 photoresist layer with the following deposition of Ti (10 nm) and Ru (150 nm). All metallic layers are magnetron sputtered. The lift-off process is carried out to make the pattern of electrodes in Ru, then the Ti/Al layer is chemically etched away via the mask of Ru. The concentric design of the electrodes provides the highest membrane deflection compared to other possible configurations.

The side walls of the chamber and filling channels (100 μm wide) are made in 16 μm thick layer of SU-8 3005 photoresist. The chamber is sealed by a flexible polydimethylsiloxane (PDMS) membrane with a thickness of 30 μm. To do the sealing a separate Si wafer is covered by a 30 μm thick PDMS layer. PDMS Sylgard 184 by Dow Corning is used with a base and curing agent mixed at 10:1 ratio. A thick (4.7 mm) auxiliary block of PDMS with a circular opening (8 mm in diameter) located in the center is bonded to the 30 μm layer of PDMS. Due to weak adhesion between Si and PDMS the block covered by the 30 μm membrane can be easily separated from the wafer. The procedure has been described in detail^35^. Finally, the block with the membrane is bonded to the SU-8 layer using the method proposed in^38^.

To test degradation of different electrode materials, special samples containing the concentric electrodes have been fabricated. These samples did not include the chamber and they were tested in a Petri dish with a sufficient amount of electrolyte. For Ru, Ti, and Au a 100 nm thick top layer has been deposited on 500 nm Al layer. For Cu the electrodes had a thickness of 500 nm (deposited on 10 nm Ti adhesive layer) and did not include the Al sublayer.

### Characterization

A molar solution of sodium sulfate in distilled water is used as an electrolyte. The conductivity of the electrolyte measured with Mettler Toledo SevenMulty is $\sigma _0=15$ S/m at room temperature 25$^\circ$C and increases with temperature with the thermal coefficient $\alpha =0.024$ K$^{-1}$. The chamber is filled with the electrolyte via the openings in the PDMS block connected with the channels in the SU-8 layer.

The actuator is driven by a homemade computer-controlled generator. The voltage pulses applied to the contact pads are generated by STM32F051R8T6 microcontroller using direct digital synthesis method and amplified 20 times by a built-in class AB power amplifier described previously^39^. The driving voltage and current flowing through the electrodes are registered by a 4-channel USB oscilloscope 5444D. Two other channels are used to capture the output signals of the homodyne quadrature interferometer that is used to measure the membrane deflection. The optical scheme of the interferometer and the way to calculate the deflection from the raw signal are described in^35^. For the interferometric measurements a 20 nm thick layer of Al is deposited onto the membrane to increase its reflectivity.

Degradation of the electrodes is inspected visually using an optical microscope equipped by a camera Moticam 1SP. Chemical composition is determined using an energy dispersive X-ray spectrometer Oxford Instruments INCA x-ACT mounted into the scanning electron microscope (SEM) Zeiss Supra 40. The measurements are performed at an accelerating voltage of 6 kV. The surface of electrodes is analysed with SEM and atomic force microscope (AFM) Smart-SPM 1000 (AIST-NT). The latter is used in the tapping mode.

## Results and discussion

### Working principle

The voltage applied to the electrochemical cell in the AP process is shown in Fig. 1a for the driving frequency $f=100$ kHz. In this process the same electrode is used alternately as the cathode and as anode with a switching time in the range of microseconds. The current flowing through the cell is shown in Fig. 1b. For each pulse the time dependence of the current is well described by the function $I(t)=I_F+I_ce^{-t/\tau }$, where $I_F$ is the Faraday current during the pulse, $I_c$ is the charge-discharge amplitude, and $\tau$ is the time constant. The diffusion is too slow to limit the current on the microsecond timescale. The process is dominated by the near electrode convection and becomes limited by the diffusion on the timescale $t\sim L^2 D\sim 1$ s, where $L=50\ \mu$m is the distance between the electrodes and $D\sim 10^{-9}$ m$^2$/s is the electrolyte diffusion coefficient. Presence of the Faraday current means that the gases are generated, however, no bubbles strongly scattering visible light are formed^10^. In contrast with the normal electrolysis, the high density Faraday current generates H$_2$ and O$_2$ NBs above the same electrode and a cloud of NBs covering the electrodes is routinely visualized due to significant variation of the refraction index of the solution^17^. The size of NBs has been measured with the dynamic light scattering^39^ as 60–80 nm.

The chip prepared for testing is shown in Fig. 1c. A zoomed view of the chamber is presented in panel d and the cross section of the device is shown schematically in panel e. When the pulses are applied to the electrodes continuously, $I_F$ increases as shown in Fig. 1f.

The current increase with time is explained by heating of the solution. If the electrolyte in the near electrode region is heated, the Faraday current grows owing to the increase of the conductivity as $I_F(T)=I_0\left[ 1+\alpha (T-T_0)\right]$, where *T* is the current temperature, $T_0$ is the room temperature, and $I_0=I_F(T_0)$. This dependence can be used as a thermometer similar for the resistance thermometer. However, the growth of the current cannot be explained by the Joule heating because the effect depends on the frequency of the driving pulses as one can see in Fig. 1f. The temperature dependence of the current shows that the liquid in the chamber is heated on $18^{\circ }$ and $24^{\circ }$C for $f=100$ and 500 kHz, respectively. The heating is actually originates from the spontaneous reaction between H$_2$ and O$_2$ NBs^12^. The same reaction is responsible for a rapid reduction of pressure in the chamber when the pulses are switched off.

**Figure 2:**
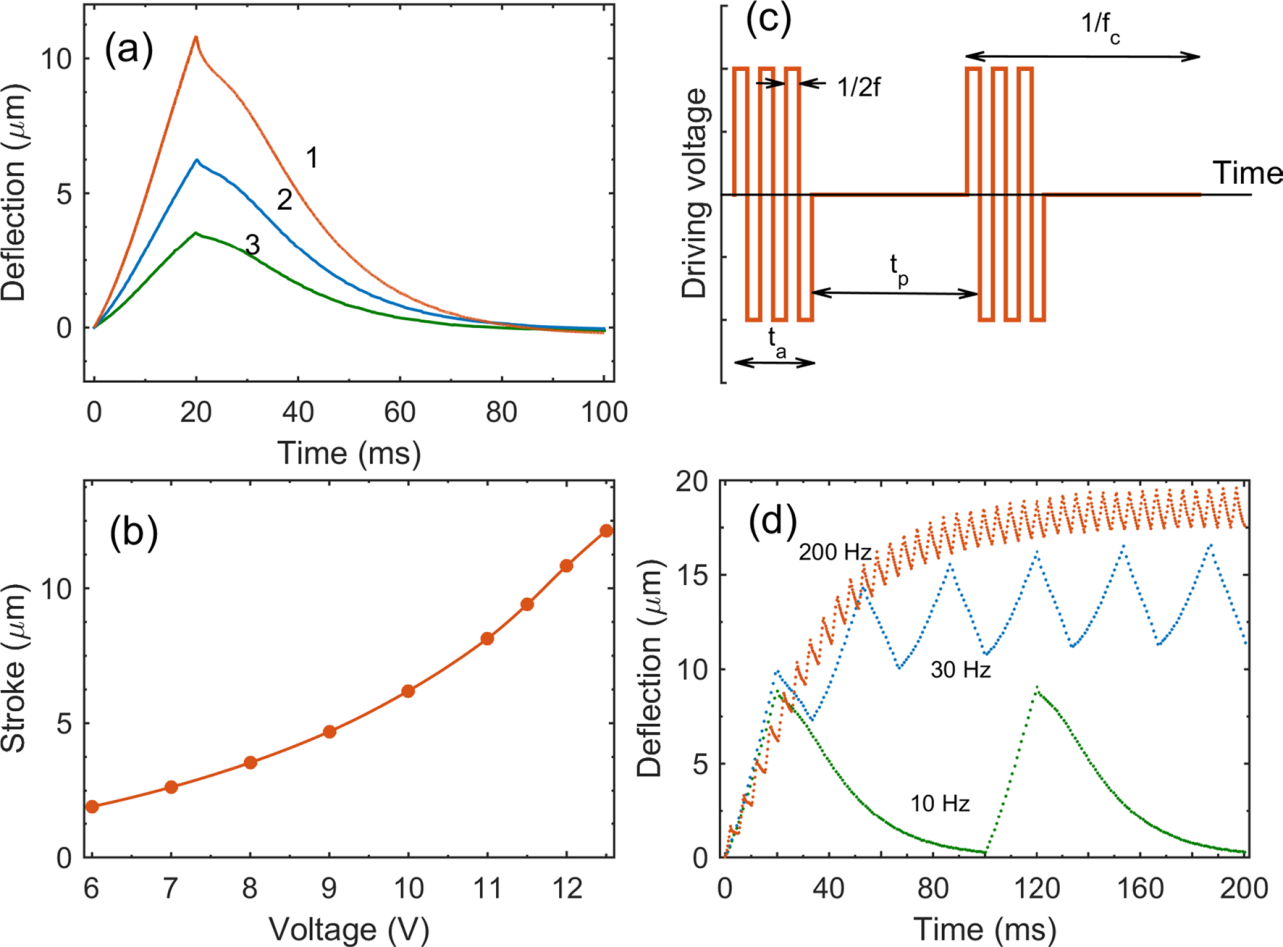
Characterization of the actuator at $f=500$ kHz. (**a**) Deflection of the membrane by a 20 ms series of pulses. The graphs show one period of operation of the actuator at cyclic frequency $f_c=10$ Hz with the amplitude of the AP pulses 12 V (1), 10 V (2), and 8 V (3). (**b**) Maximum deflection of the membrane as a function of the amplitude of the driving pulses. (**c**) Scheme of the voltage powering the actuator in the cyclic operation regime. The important characteristics are shown. (**d**) Cyclic operation of the actuator for the frequencies $f_c=10, 30,$ and 200 Hz. For 10 and 30 Hz the active time is 20 ms. For $f_c=200$ Hz the active time is 3 ms and the amplitude of pulses is $U=14.2$ V.

Figure 2a shows the deflection of the membrane as a function of time when a series of AP pulses 20 ms long is applied to the Ru electrodes. During this time the membrane is pushed up by the NBs produced in the chamber. When the pulses are switched off, the NBs with different gases recombine via the spontaneous combustion. The bubbles containing the same gas are protected from merging due to repulsion of negative charges adsorbed on their surfaces^40^, but still they merge slowly by the Ostwald ripening mechanism. When two bubbles are separated by a liquid membrane, they can exchange the content by diffusion with a rate, which is proportional to the concentration difference of one and the same gas in the bubbles. For the bubbles with the same gas this difference is small, but for the bubbles with different gases the difference is maximum and oxygen from one bubble will diffuse to the bubble with hydrogen and vice versa. As the result both bubbles will disappear in the combustion reaction.

All the gas produced in 20 ms disappears from the chamber in 80 ms. Thus, for given geometrical characteristics of the chamber and parameters of the driving pulses the device can perform an up-and-down cycle in 100 ms. The stroke of the membrane increases with the amplitude of the pulses as shown in Fig. 2b. When the amplitude of the pulses is above 12.5 V, the deflection becomes large but poor controlled because of the formation of short-lived microbubbles. These bubbles appear when the density of NBs is so high that many NBs merge simultaneously and explode as explained in^17^.

For practical applications the actuation has to work in a cyclic regime when the membrane is moving up and down with a certain cyclic frequency $f_c$. For this regime the driving pulses at the driving frequency (*f*) are applied in series repeated with the frequency $f_c$ as shown in Fig. 2c. During the active time $t_a$ the actuator is powered by the high-frequency *f* alternating polarity pulses, then during the passive time $t_p$ no pulses are applied. After the period $t_c=t_a+t_p$ the cycle is repeated so that the cyclic frequency is $f_c=t_c^{-1}$. Video 1S demonstrates the cyclic operation of the actuator at 10 Hz. It was recorded at 19 frames per second. The driving pulses had the amplitude $U=9$ V and frequency $f=500$ kHz. One can see that the membrane is going up and down, but no bubbles, which are able to scatter the visible light, are formed in the chamber. The gas collected in NBs can be the only reason for a stroke of about $5\ \mu$m. For example, homogeneous heating of liquid on $20^{\circ }$C can only be responsible for a stroke of $0.2\ \mu$m.

Simple estimates based on the Faraday and ideal gas laws support the description presented above (see Supplementary information for details). The current at the amplitude $U=10.5$ V during the 20 ms series as in Fig. 2a is $I_F=60.5$ mA. Then the total number of gas molecules produced during the series is $N_g=5.67\times 10^{15}$. Roughly only a thousandth part of these molecules can be dissolved in the chamber. At normal conditions the excessive gas would fill the volume that is 75 times larger than the volume of the chamber $V_{ch}=3.14\times 10^{-12}$ m$^3$. Although at $f=500$ kHz the bubble size has not been directly measured, we estimate the radius of NBs as $r=25$ nm extrapolating the results of the dynamic light scattering^39^. Taking into account the Laplace pressure in a NB one finds the number of molecules in a bubble as $N_{NB}=0.93\times 10^5$. In the NBs the produced gas molecules are packed more efficiently and all the gas would take the volume $\Delta V_{id}=3.98\times 10^{-12}$ m$^3$ in the ideal case when no reaction between the gases during the series. The realistic volume taken by the NBs is $\Delta V=0.73\times 10^{-12}$ m$^3$ as estimated from the stroke of the membrane $d=7.4$ μm. It means that only 18% of the gas survive in the form of separate H$_2$ and O$_2$ NBs. All the rest NBs recombine during the series in the near electrode region where the concentration of NBs is the highest as was observed in^10^. The concentration of the survived NBs averaged over the volume $V_{ch}+\Delta V$ is estimated as $n_{NB}=2.87\times 10^{21}$ m$^{-3}$. The average distance between the centers of NBs is $a\simeq n_{NB}^{-1/3}=70$ nm. When the pulses are switched off there is a high probability for H$_2$ and O$_2$ NBs to approach to a distance of a few nanometers and recombine due to diffusion of the gases. This process provides the mechanism for a fast disappearance of the gas from the chamber.

Even though a detailed mechanism of the spontaneous combustion in NBs is not known, it has to be related to the formation of radicals in NBs. If one assumes that H$_2$ molecules are able to dissociate on the inner surface of NB, then there is a reaction path, transforming a mixture of H$_2$ and O$_2$ gases into water, that does not require a high activation energy needed for normal combustion^41^. In this sense the reactions that occur in NBs can be called “cold combustion”. The existence of such a path has been confirmed independently using reactive molecular dynamic simulations^42^.

Although for given geometrical characteristics the optimal cyclic frequency is $f_c=10$ Hz, the actuator can perform faster. In Fig. 2d cyclic operation with frequencies 10, 30, 200 Hz is shown. For $f_c=30$ Hz the pulses are switched on for the same 20 ms (active time), but switched off for a shorter period of 13.3 ms (passive time). The same amount of gas as for $f_c=10$ Hz is produced, but the passive time is too short for all the gas to recombine. The survived gas is collected in the chamber increasing the average concentration of NBs until a steady state is established. In the equilibrium the membrane has an offset and the amplitude of oscillations 5.1 μm is smaller than that 8.8 μm for $f_c=10$ Hz. The effect of saturation of the chamber with NBs is visible especially well for $f_c=200$ Hz. In this case the active time is 3 ms and the passive one is 2 ms. The oscillation amplitude is 1.9 μm with the offset of 15 μm, however, one has to keep higher amplitude of the pulses 14.2 V instead of 11 V as for $f_c=10$ or 30 Hz. With further increase of the cyclic frequency the offset continues to grow, but the oscillation amplitude approaches a fixed value of 1.5 μm. The maximum frequency we have tested was $f_c=450$ Hz.

### Degradation of electrodes

For the frequency of pulses $f=500$ kHz the average density of the Faraday current is $J_F=I_F/\pi R^2=300{-}400$ A/cm$^2$, where $R=75$ μm is the radius of the central electrode. Even higher values of $J_F$ are reached locally near the edges of the electrodes. For these high current densities fast degradation of the electrode materials occurs, however, the reasons for the degradation can be different. A detailed investigation has been performed for platinum electrodes^36^. It was concluded that Pt electrodes are destroyed mechanically by a local energy deposition due to the reaction between NBs near the electrode surface. Investigation of Ti electrodes revealed different mechanism of the degradation^37^. In this case the growth of titanium oxide on the electrode surface is responsible for the reduction of the Faraday current during the first minute of the AP process.

**Figure 3:**
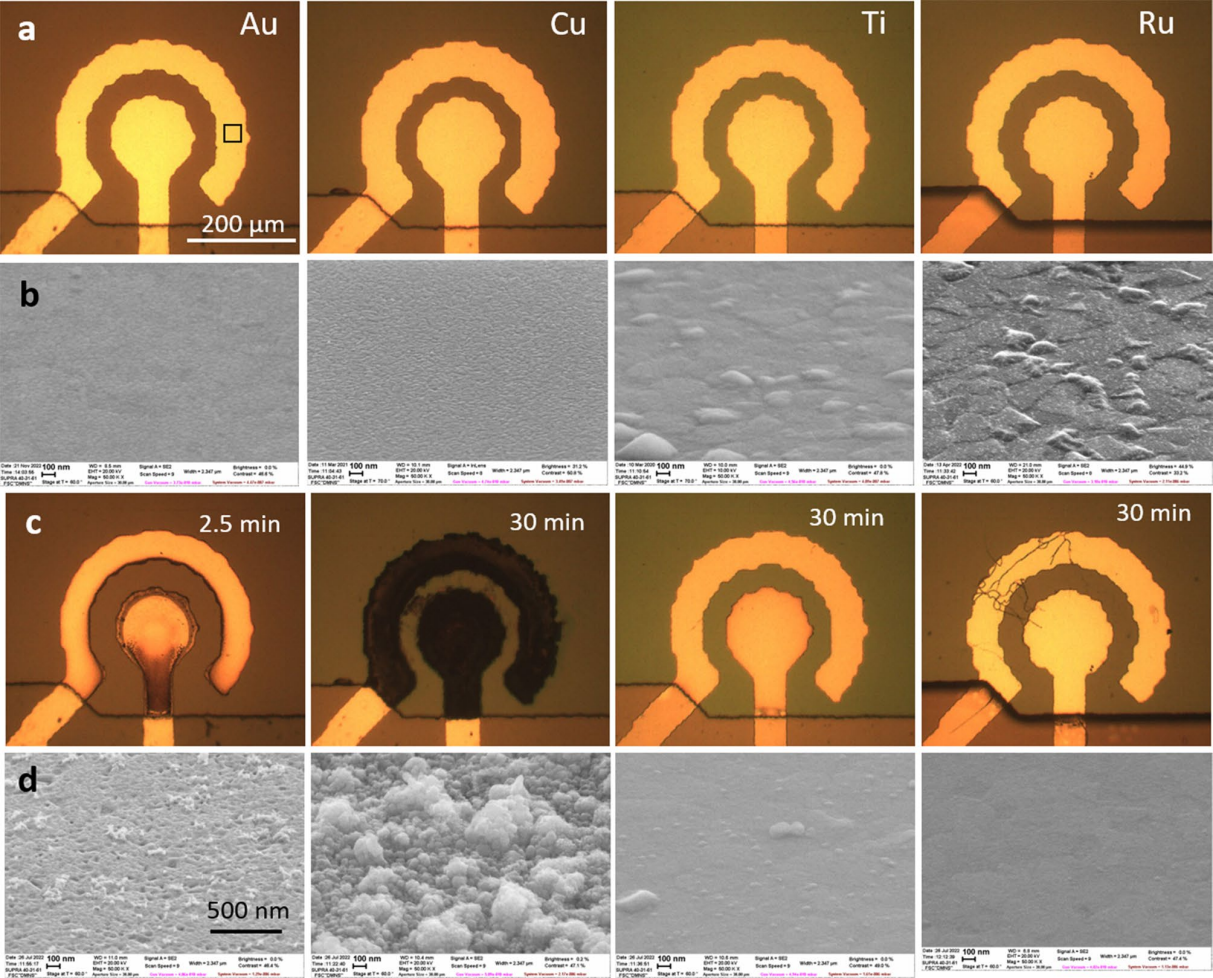
Degradation of electrodes from different materials. (**a**) (row) Optical images of the electrodes from different materials before the test. (**b**) (row) SEM images of electrodes before the test. Specific roughness of Au, Ti, and Ru because of the underlying Al. (**c**) (row) Optical images of the electrodes after application of the AP pulses with $f=500$ kHz and $U=7$ V during 2.5 min for Au and 30 min for other materials. (**d**) (row) SEM images after the test of the rim electrode in the place marked by the black rectangular for Au. All SEM images are made at a stage angle of 60$^\circ$.

We did additional analysis of the electrodes made of different materials but having the same geometry. Some results are shown in Fig. 3. The row a shows the optical images and the row b SEM images of electrodes before the test, the rows c and d show the images after the test. The Au electrodes are destroyed fast and the process is accompanied by the current reduction. The SEM images demonstrate the features very similar to those for Pt. In the places, where the current density is a relatively small, the characteristic pits and nanoparticles can be seen on the surface. In the places with higher current density the nanoparticles are piled chaotically. The Cu electrodes are also actively degrade but survive longer time because of larger thickness of the Cu layer. During 30 min the surface becomes well developed and demonstrate debris between the electrodes. Clear similarity between Pt, Au, and Cu can be seen in Supplementary Fig. 1S, where SEM images near the edge of the electrodes are shown. We conclude that for all these relatively soft materials (Mohs hardness is 3.5, 3.0, and 2.5 for Pt, Cu, and Au, respectively) the degradation occurs by the same mechanism as for Pt: explosions of NBs near the surface generate pits and nanoparticles, which are collected in piles with time.

**Figure 4:**
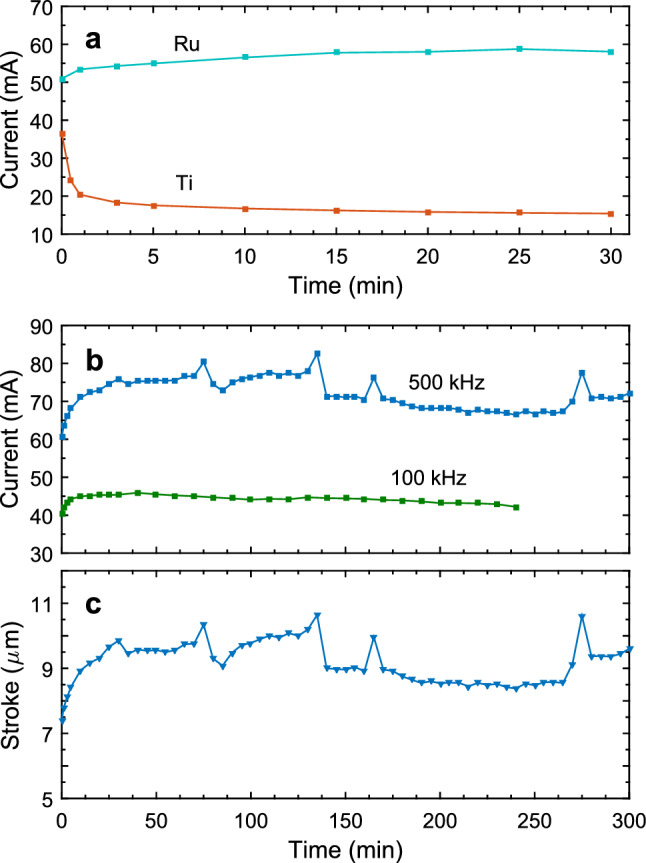
Lone-time performance of the electrodes. (**a**) Current as a function of time through Ru and Ti electrodes shown in Fig. 3. ( **b**) Long-time test of the actuator with Ru electrodes. The current as a function of time is shown for the parameters $f_c=10$ Hz, $U=10.5$ V, and $f=500$ kHz (blue curve) and $f_c=10$ Hz, $U=8.1$ V, and $f=100$ kHz (green curve). (**c**) Stroke of the membrane for the test with $f=500$ kHz. The membrane movement reproduces all fluctuations of the current.

A different situation is observed for harder materials such as Ti and Ru (Mohs hardness is 6 and 6.5, respectively). After 30 min processing Ti electrodes becomes darker due to oxide formation^37^. The current drops significantly in the first minute of the process. The SEM image demonstrates formation of hills, which are related to the relaxation of the local stresses due to growing oxide. Near the edge of the electrodes these hills form a continuous net. A strikingly different situation is observed for Ru. On SEM images one can notice very small rare hills, which do not form a net even at the edges of the electrodes. In contrast with Ti the current is not reduced but grows with time as one can see in Fig. 4a, where the current as a function of time is presented for both Ti and Ru. In comparison with softer materials no signs of mechanical destruction can be seen on these harder electrodes.

It is possible to separate the third class of electrode materials. One could expect that the hardest materials like Cr or W will suit the best for the electrodes, however, it is definitely not the case. Chromium is going very fast (in seconds) to the solution because of anodic dissolution^43^. The dissolution occurs also for W^44^ but at a lower rate. This property makes impossible to use these materials as electrodes.

From the analysis above one can conclude that Ru is the most promising material for the electrodes, which is able to withstand the high current density of the AP electrochemical process.

### Long-time performance of the actuator

Ruthenium is a somewhat harder material on the Mohs scale than titanium, but in contrast with TiO$_2$ ruthenium oxide has a metallic conductivity. Dissolution of Ru is widely investigated in relation to the electrochemical production of hydrogen^45^, but stability of this material in the AP electrochemical process has not yet been tackled. In this relation we analyse the long-time performance of the actuator with ruthenium electrodes.

The actuator has been tested in a 5 h run for cyclic operation at $f_c=10$ Hz with the driving pulses $U=10.5$ V and $f=500$ kHz. The current and the stroke of the membrane are shown in Fig. 4b,c, respectively. The current is averaged during the active time of the cycle. It has to be noted that for Ru electrodes the current starts to grow with the operation time reaching the steady state in half an hour. This current increase is explained by heating of the substrate that happens on a longer timescale than the heating of liquid in the chamber responsible for the current increase on the short-time scale as in Fig. 1f. Some long-time fluctuations of the current are observed during the steady state, but the current is kept at the same level. The origin of these fluctuations is not quite clear, but the amplitude of the fluctuations varies from sample to sample. The stroke reproduces all the details of the current behaviour.

**Figure 5:**
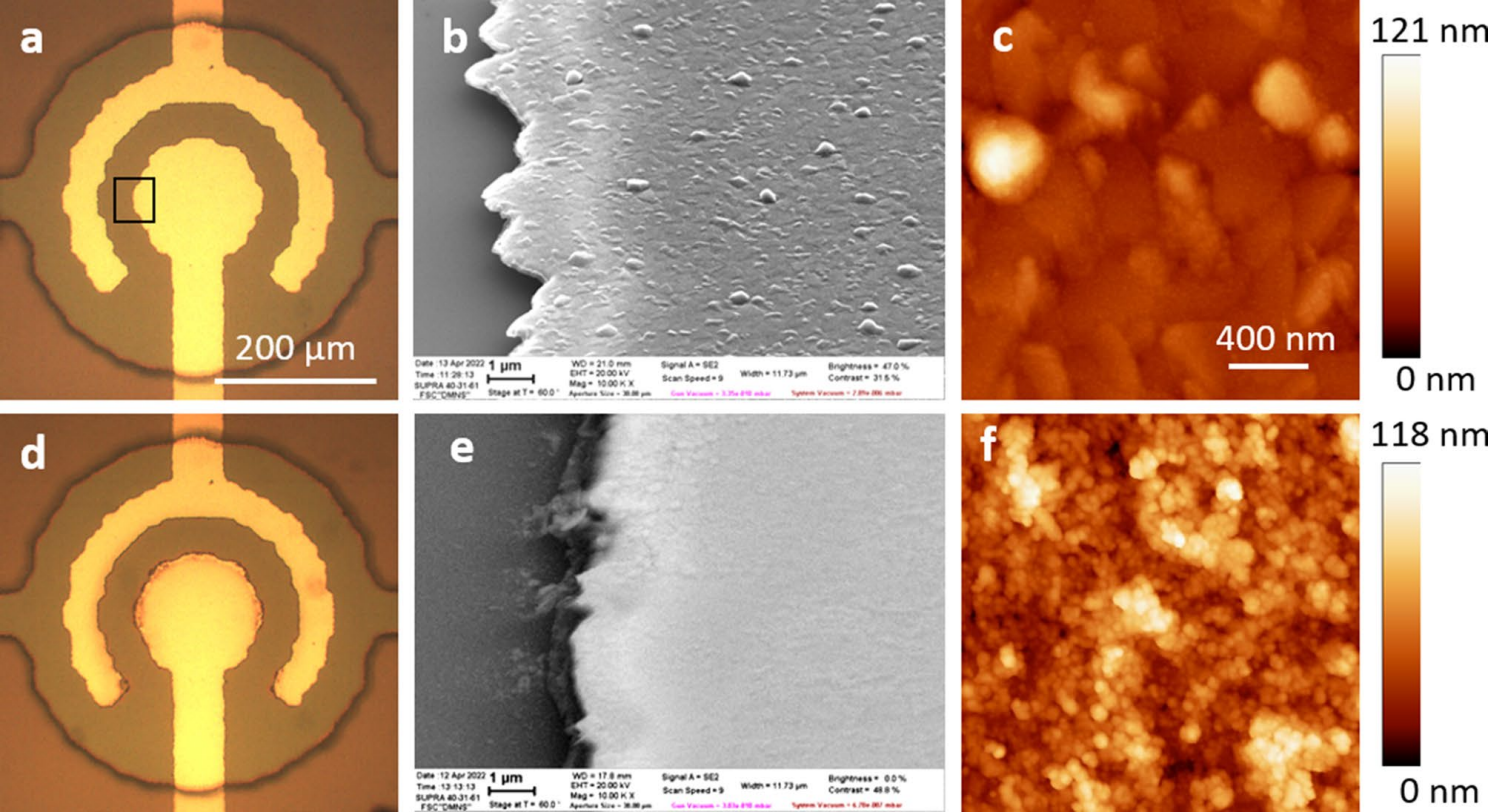
Surface of the electrodes before and after the long-time test. (**a**) Optical image of the chamber and electrodes before the test. The black rectangular indicates the area investigated with SEM and AFM. (**b**) SEM image of the edge of the central electrode. (**c**) AFM image made in the same area as the SEM image in (**b**). The color axis is shown on the right of the image. (**d**–**f**) Show the same as (**a**–**c**) but after 5 h test with the parameters $f_c=10$ Hz, $U=10.5$ V, and $f=500$ kHz.

Comparison of the electrode surfaces before and after 5 h operation at $f_c=10$ Hz is shown in Fig. 5 for the frequency of the driving pulses $f=500$ kHz (Fig. 5a–c before and d–f after the test). Before the test the electrode surface demonstrates relatively large grains with a size of 100–300 nm with characteristic hillocks that are related to the underlying layer of Al. With AFM imaging on top of the large grains one can see smaller grains of about 30 nm in size that can be identified as the grains of ruthenium. The optical image of the electrodes after the test has a slightly darker edge of the central electrode. Although the peak-to-peak roughness of the surface is the same as that before the test, the topography of the surface changes significantly: the large grains disappear, but the smaller ones become larger. It indicates the formation of ruthenium oxide on the surface. The presence of additional oxygen in the surface layer is supported by the energy-dispersive X-ray (EDX) analysis. Before the test oxygen is homogeneously distributed over the central electrode with an amount of 8.8 atomic percent (at.%). After the test the amount of oxygen is 21.5 and 13.0 at.% at the edge and in the center of the electrode, respectively. Inhomogeneous distribution of the oxide results in darkening of the edge of the electrode in Fig. 5d. No signs of degradation of the electrodes can be seen in Fig. 5e,f.

**Figure 6:**
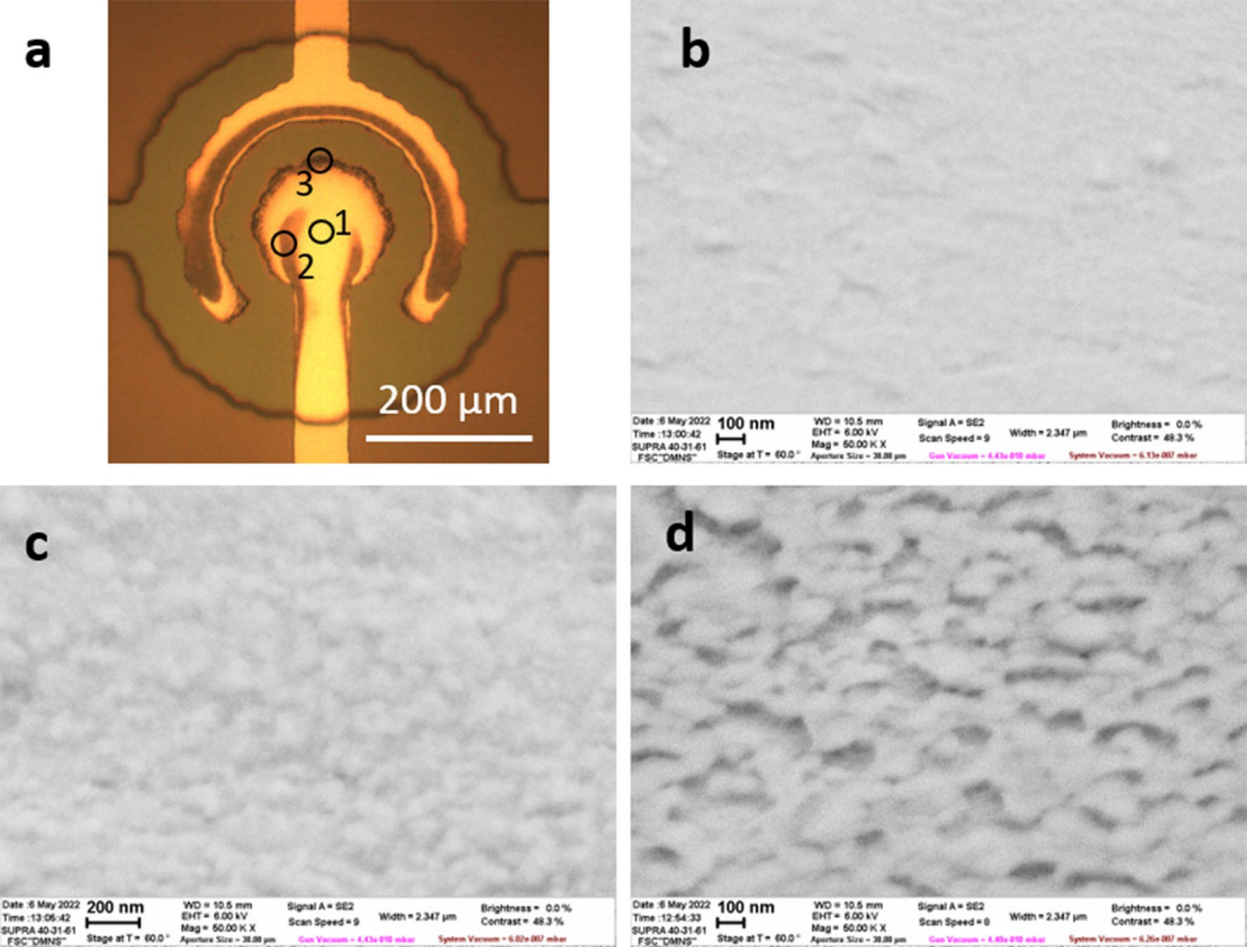
Electrodes after the test with the driving frequency $f=100$ kHz. (**a**) Optical image of the electrodes after 4 h test at $f_c=10$ Hz, $U=8.1$ V. The black circles mark the areas investigated with SEM. (**b**–**d**) SEM images taken in the areas 1, 2, and 3, respectively. The stage angle is 60$^\circ$.

The situation is different for the lower driving frequency $f=100$ kHz. After 4 h of continuous work with the amplitude of the driving pulses $U=8.1$ V at cyclic frequency $f_c=10$ Hz the electrodes demonstrate a significant darkening as can be seen in Fig. 6a. The increase of the amplitude above 8.5 V is not possible because of the formation of exploding microbubbles and poor control of the membrane deflection^17^. The current during the 4 h test is shown in Fig. 4b by the green curve. It increases in the process of the initial heating, but then it goes slowly down. The decrease of the current is a sign of the electrode degradation. More information on the degradation one can get from SEM images Fig. 6b–d collected in the areas 1, 2, and 3 as marked in Fig. 6a by the circles. From 1 to 3 the surface becomes more and more developed. It correlates also with the amount of oxygen determined by the EDX analysis that increases as 14.5, 22.3, and 37.7 at.% for the positions 1, 2, and 3, respectively. It is possible to conclude that with the reduction of the driving frequency the electrodes start to degrade. We relate this effect with the increase of the NBs size when the driving frequency decreases. For larger bubbles the local combustion releases more energy having stronger impact on the surface of the electrodes.

### Discussion

The reduction of the bubble size with the increase of the driving frequency agrees well with both expectations and observations. This dependence has been observed with the dynamic light scattering method^39^, where the average bubble size has changed from 80 nm at $f=150$ kHz to 60 nm at $f=325$ kHz. The stronger heating of liquid with increasing frequency, as shown in Fig. 1f, is also explained by the reduction of the bubble size. The diffusion layer above the electrodes is strongly supersaturated with the gases and its thickness scales with the frequency as $f^{-1/2}$. The NBs are nucleated in this layer and then pushed out by the new NBs generated in the next period. The thinner the diffusion layer the more H$_2$ and O$_2$ NBs are close enough to merge and produce the heat. Thus, the near electrode region, where the current density is the highest, is heated stronger for the higher driving frequency. The bubble size is also responsible for the increase of the threshold amplitude of the pulses. This amplitude corresponds to the density of NBs, when they nearly touch each other. In this case the simultaneous merging of many NBs results in the formation of short-lived microbubbles. The larger is the NB size the smaller amplitude *U* needed to reach the critical density.

Operation of the actuator at high cyclic frequencies is very important for practical applications. For the investigated device the preferable frequency is not more than 10 Hz; otherwise, the middle position of the membrane will be lifted up that is not always possible in normal operation. For fast recombination of H$_2$ and O$_2$ NBs the average distance between the bubbles in the chamber has to be as short as possible. One can expect that for a given height of the chamber there is an optimal frequency $f_c$. The smaller the height of the chamber the higher the operation frequency. For the chamber covered with a soft PDMS membrane it is difficult to reduce the height of the chamber, but it is possible for stiffer materials. For example, for the chamber with a height of $5\ \mu$m covered by a 150-nm thick SiRN membrane the recombination time as short as $200\ \mu$s has been demonstrated^12^. Thus, we believe that the cyclic frequency of the actuator with Ru electrodes can be significantly increased without offset of the membrane.

## Conclusion

The acceleration of chemical reactions on water microdroplets and formation of radicals in nanobubbles have been discovered in the last decade. The observed chemical processes demonstrate unusual activity of the gas-water interface for objects with a high surface-to-volume ratio. Although the reason for this activity is not clear, it is possible to use the phenomenon for practical purposes as demonstrated in this paper. We fabricated an electrochemical actuator that generates NBs producing pressure in a closed chamber and annihilate the gases in the spontaneous combustion of hydrogen and oxygen in NBs relieving the pressure in milliseconds. In comparison with the existing electrochemical actuators the response time is reduced orders of magnitude. However, the main problem of such an actuator is the degradation of electrodes. All but one tested materials degrade in a minute or so of continuous operation of the actuator because of a high current density in the AP electrochemical process. It was found that only ruthenium electrodes have no signs of degradation in the long-term test. The longevity of Ru is due to a unique combination of high hardness and metallic conductivity of ruthenium oxide. Nevertheless, even this material started to degrade, when the frequency of the driving pulses was reduced to $f=100$ kHz. This effect was related to the increase in NB size and a larger release of energy from the explosions of NBs near the surface than that at $f=500$ kHz.

The actuator breaks prejudices that the gas-water has no catalytic properties and that the combustion of hydrogen-oxygen mixture is not possible in volumes smaller than a few microliters. In this sense, the realization of the actuator has a fundamental significance. On the other hand, thanks to its small size the actuator can be used for practical purposes as an engine driving autonomous microfluidic devices in medical and biological applications. Moreover, the dimensions of the actuator can be scaled down further, if one will use thinner and stiffer membrane, for example, made of silicon nitride as that used in^12^.

## Supplementary Information

Supplementary Information.

## Data Availability

All data generated or analysed during this study are included in this published article and its supplementary information files.
