# Supplementary material for: Nanoreactors in action for a durable microactuator using spontaneous combustion of gases in nanobubbles
Source: Sci Rep. 2022 Dec 3;12:20895. doi: 10.1038/s41598-022-25267-2 (PMC9719487; doi:10.1038/s41598-022-25267-2)
Supplement: Supplementary file 1 — Supplementary Information. [file 41598_2022_25267_MOESM1_ESM.pdf]

Supplementary information

Nanoreactors in action: a durable microactuator using spontaneous combustion of gases in nanobubbles

Ilia V. Uvarov

Valiev Institute of Physics and Technology,

Yaroslavl Branch, Russian Academy of Sciences,

Universitetskaya 21, Yaroslavl, 150007, Russia

Vitaly B. Svetovoy*

Frumkin Institute of Physical Chemistry and Electrochemistry,

Russian Academy of Sciences, Leninsky prospect 31 bld. 4, 119071 Moscow, Russia

* Corresponding author: v.svetovoy@phyche.ac.ru

Supplementary figure

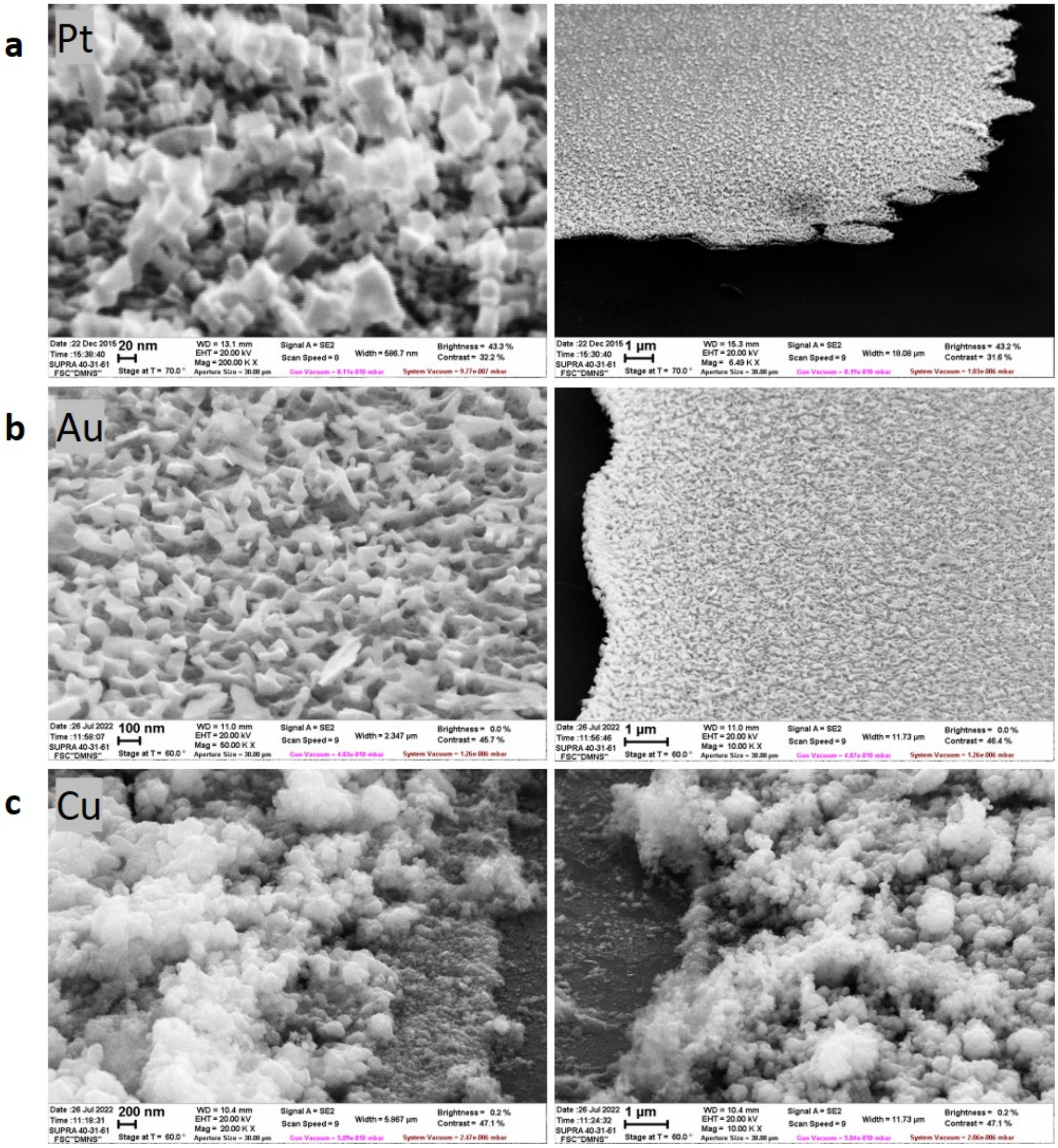

FIG. 1S. **Comparison of Pt, Au, and Cu degradations.** **a** (row) SEM images of platinum near the edge of electrode at two different magnifications; the process time is 1 min. **b** (row) The same for gold; the process time is 2.5 min. **c** (row) The same for copper; the process time is 30 min.

Estimate of the density of nanobubbles

The Faraday current measured during the AP process allows determination of a number of helpful parameters that describe NBs in the working chamber. The estimates below are based on the Faraday law and on the ideal gas law. The Laplace pressure in NBs is not high enough to consider the nonideality corrections for the gas law.

As an example for calculations we choose the amplitude of the driving pulses $U = 10.5$ V at frequency $f = 500$ kHz. If the AP electrochemical process is driven cyclicly, the current grows with time. We do the estimates for the very first cycles when the current is the smallest. For these cycles it was found that the Faraday current during the active time is equal to $I_F = 60.5$ mA. For the cyclic frequency $f_c = 10$ Hz the series of pulses lasts for $\tau = 20$ ms. The total number of gas molecules (H_2 and O_2), generated for this time, is

$$N_g = \frac{3}{4e} I_F \tau \approx 5.67 \times 10^{15}, \tag{1}$$

where e is the absolute value of the electron charge. Keeping in mind that the volume of the chamber is $V_{ch} = \pi R^2 h \approx 3.14 \times 10^{-12}$ m^3 ($R = 250$ μm, $h = 16$ μm) and using the ideal gas low one can find that this gas at normal conditions would take the volume of $75.4 V_{ch}$.

Only a small part of the gas molecules can be dissolved in the electrolyte. The saturated concentrations are 4.7×10^{23} m^{-3} and 7.7×10^{23} m^{-3} for hydrogen and oxygen, respectively. Then the total number of the gas molecules that can be dissolved in the chamber is $N_s = 3.9 \times 10^{12}$ that is less than 0.1%. Moreover, these molecules can dissolve in the chamber only after the first cycle. During the second cycle the solution will be already saturated with hydrogen and oxygen.

Because the chamber is closed all the produced gas has to be packed in some way so that the membrane would raise only on the measured value $d = 7.4$ μm. The PDMS membrane is very soft and its shape is better described by a paraboloid. Then the increment of the volume of the chamber is

$$\Delta V = \frac{\pi R^2 d}{2} \approx 0.73 \times 10^{-12} \text{ m}^3. \tag{2}$$

If the NBs have the average radius r, then the number of gas molecules in one bubble with an account of the Laplace pressure is

$$N_{NB} = \frac{P_a + 2\gamma/r}{kT} \left(\frac{4\pi r^3}{3} \right), \tag{3}$$

where $P_a \approx 10^5$ Pa is the atmospheric pressure, kT is the temperature in the energy units, and $\gamma \approx 0.072$ J/m^2 is the surface tension of water.

The experimental value of the bubble radius r at a pulses frequency of 500 kHz is not known. In [39] (main text reference) this parameter has been measured at $f = 150$ kHz as $r = 40$ nm and at $f = 325$ kHz as $r = 30$ nm. Since it is expected that the radius decreases with the increase of frequency, we extrapolate the radius to higher frequencies as $r = (a + bf)^{-1}$, where a and b are the parameters. In this way we find $r \approx 25$ nm at $f = 500$ kHz keeping, however, the uncertainty in this value as ± 5 nm.

For this bubble size from (3) one finds the number of molecules in one bubble as

$$N_{NB} = 0.93 \begin{array}{c} +0.41 \\ -0.34 \end{array} \times 10^5. \tag{4}$$

If all the gas generated for 20 ms would left in the chamber (ideal case), then the volume increment would be

$$\Delta V_{id} = \left(\frac{N_g}{N_{NB}}\right) \frac{4\pi r^3}{3} = 3.98 \begin{array}{c} +0.81 \\ -0.76 \end{array} \times 10^{-12} \text{ m}^3. \tag{5}$$

This value is considerably larger than the observed increment (2). The reason here is the following. The recombination reaction occurs not only after but during the gas generation as well. Moreover, near the electrodes the concentration of NBs is the highest and the reaction rate is larger there than that in the chamber volume. Thus, only part of the produced gas is collected in the chamber in the form of H_2 and O_2 NBs, but the rest of the gas disappears in the reaction during the active time. The part of the survived gas is estimated as

$$\frac{\Delta V}{\Delta V_{id}} = 18 \begin{array}{c} +5 \\ -3 \end{array} \%. \tag{6}$$

It means that roughly only fifth part of the generated gas is able to produce useful work.

Now we can estimate the concentration of NBs generated by a series of pulses 20 ms long in the augmented volume of the chamber. It can be presented in the form

$$n_{NB} = \frac{N_g/N_{NB}}{V_{ch} + \Delta V} \left(\frac{\Delta V}{\Delta V_{id}}\right) = \frac{\Delta V}{V_{ch} + \Delta V} \left(\frac{4\pi r^3}{3}\right)^{-1} = 2.9 \begin{array}{c} +2.7 \\ -1.2 \end{array} \times 10^{21} \text{ m}^{-3}. \tag{7}$$

It is rather high concentration since the average distance between the bubbles is smaller than the bubble size. The average distance between the bubbles we estimate as

$$a = n_{NB}^{-1/3} = 2.81r. \tag{8}$$

This distance is proportional to the bubble radius and the distance between the walls of neighboring bubbles $a - 2r = 0.81r$ varies between 16 and 24 nm in dependence of r.
